# Biochemical and biophysical characterization of four EphB kinase domains reveals contrasting thermodynamic, kinetic and inhibition profiles

**DOI:** 10.1042/BSR20130028

**Published:** 2013-06-05

**Authors:** Ross C. Overman, Judit E. Debreczeni, Caroline M. Truman, Mark S. McAlister, Teresa K. Attwood

**Affiliations:** *AstraZeneca PLC, Alderley Park, Cheshire, SK10 4TG, U.K.; †Faculty of Life Sciences and School of Computer Science, The University of Manchester, Oxford Road, Manchester M13 9PL, U.K.

**Keywords:** EphB1, EphB2, EphB3, EphB4, kinase inhibition, protein stability, CMPD3, compound 3, DSF, differential scanning fluorimetry, DTT, dithiothreitol, Eph, erythropoietin-producing hepatocellular carcinoma, GdnHCl, guanidine hydrochloride, ITC, isothermal titration calorimetry, Ni-NTA, Ni^2+^-nitrilotriacetate, PTP1B, protein tyrosine phosphatase 1B, RTK, receptor tyrosine kinase, SEC, size-exclusion chromatography, TCEP, tris-(2-carboxyethyl)phosphine, TEV, tobacco etch virus, TFA, trifluoroacetic acid

## Abstract

The Eph (erythropoietin-producing hepatocellular carcinoma) B receptors are important in a variety of cellular processes through their roles in cell-to-cell contact and signalling; their up-regulation and down-regulation has been shown to have implications in a variety of cancers. A greater understanding of the similarities and differences within this small, highly conserved family of tyrosine kinases will be essential to the identification of effective therapeutic opportunities for disease intervention. In this study, we have developed a route to production of multi-milligram quantities of highly purified, homogeneous, recombinant protein for the kinase domain of these human receptors in *Escherichia coli*. Analyses of these isolated catalytic fragments have revealed stark contrasts in their amenability to recombinant expression and their physical properties: e.g., a >16°C variance in thermal stability, a 3-fold difference in catalytic activity and disparities in their inhibitor binding profiles. We find EphB3 to be an outlier in terms of both its intrinsic stability, and more importantly its ligand-binding properties. Our findings have led us to speculate about both their biological significance and potential routes for generating EphB isozyme-selective small-molecule inhibitors. Our comprehensive methodologies provide a template for similar in-depth studies of other kinase superfamily members.

## INTRODUCTION

The Eph (erythropoietin-producing hepatocellular carcinoma) B receptors (EC 2.7.10.1) constitute one-third of the ephrin RTK (receptor tyrosine kinase) subfamily; they are type I transmembrane proteins, and form the largest grouping within the tyrosine kinase family [1]. In warm-blooded vertebrates, the Eph subfamily consists of 15 members [2], 14 of which are present in humans [3]: namely, EphA1–A8, A10, EphB1–B4 and B6. The EphA and EphB receptors recognize and interact with their respective ligands, the ephrins, which are found on the surface of neighbouring cells, through highly selective, nanomolar affinity interactions [4]. The interactions between ephrins and Eph receptors on adjacent cells give rise to cell-to-cell contacts and bi-directional intracellular signalling cascades that mediate cellular repulsion, adhesion and migration [5]. Eph receptors are also implicated in extracellular matrix attachment [6], cell boundary formation [7] and tissue morphogenesis [8], including blood vessel maturation within the cardiovascular system [9] and axonal path finding within the nervous system [10,11].

The EphB receptors and their ligands have been implicated in the progression of a variety of human cancers, a role that appears to be complex and often conflicting, depending on the type of cancer and stage of progression [12]. For example, EphB2 kinase domain inactivating mutations have been found in prostate cancer cell lines, implicating a role for EphB2 as a tumour suppressor [13]. EphB2 has also been shown to promote cell proliferation in intestinal epithelia through its tyrosine kinase activity [14], while conversely inhibiting invasive growth by a kinase-independent mechanism [15]. EphB4 epithelial up-regulation has been observed in late-stage ovarian cancer [16], while being down-regulated, and having tumour suppressor activities, in intestinal cancers [17]. EphB3 has been shown to suppress growth of a human colon cancer cell by strengthening cell–cell contacts [18], and its increased activity can suppress non-small-cell lung-cancer metastasis [19]. Conversely, elevated levels of EphB3 have been shown to have a role in the failure of contact inhibition of locomotion exhibited by certain metastatic prostate cancer cell lines overexpressing this receptor [20], an observation that results in a more invasive phenotype. The reduced expression of EphB1 has been linked with invasion and metastasis in both colorectal [21] and gastric carcinomas [22], while blocking EphB1 forward signalling may have a clinical benefit in relieving bone cancer pain [23]. The complexity and divergent nature of the many examples of Eph receptor up- and down-regulation in so many different areas of oncology has resulted in a variety of novel anti-cancer therapies; those currently under investigation include antibodies, peptides and small-molecule kinase inhibitors [24].

The development of potent and selective small molecules to modulate the activity of the EphB receptors could aid deconvolution of the complex roles of these receptors in cancer disease models, or indeed serve as therapeutic agents. Hence, this study focuses on generating a clearer understanding of the structure–function relationship of the catalytic domains of the closely related EphB receptors (EphB1– 4), excluding the more divergent, catalytically inactive EphB6 receptor [25]. In this study, we describe *Escherichia coli*-based methods for producing and isolating multi-milligram quantities of active kinase domain for the four receptors, facilitated by phosphatase co-expression. The material, generated in a homogeneous, catalytically competent state, has been further characterized by a range of biochemical and biophysical techniques in an effort to understand the interesting disparities between these important enzymes in terms of recombinant expression, *in vitro* stability, catalytic activity and inhibitor binding profiles.

## EXPERIMENTAL

All chemicals were obtained from Sigma unless otherwise stated.

### Molecular biology

Catalytic domain genes for the four EphB tyrosine kinases were synthesized *in vitro* by GENEART AG (Life Technologies); EphB1 residues 602–896 (UniProtKB/Swiss-Prot: EPHB1_HUMAN, P54762), EphB2 residues 604–898 (UniProt/Swiss-Prot: EPHB2_HUMAN, P29323), EphB3 residues 616–910 (UniProtKB/Swiss-Prot EPHB3_HUMAN, P54753) and EphB4 residues 598–892 (UniProtKB/Swiss-Prot: EPHB4_HUMAN, P54760). An EphB3 C717G mutant was also produced as a synthetic gene, created as a variant of the wild-type sequence. All sequences were codon-optimized for *E. coli* expression. Bacterial expression vectors were generated using the Gateway® Cloning System (Life Technologies); synthesized genes were sub-cloned into the Gateway®-adapted pT7#3.3 N6His *E. coli* expression vector [26]. The resultant expression vectors contained an N-terminal His_6_ tag to facilitate purification, and a TEV (tobacco etch virus) cleavage site upstream of each EphB catalytic domain: MHHHHHHGSTSLYKKAGSENLYFQGSS. An additional expression vector (pRSF1-PTP1B) for phosphatase co-expression was also constructed. pRSF1-PTP1B contained a single copy of the human PTP1B (protein tyrosine phosphatase 1Beta) protein (UniProtKB/Swiss-Prot: PTN1_HUMAN, P18031, residues 1-288) inserted into the pRSF-1b plasmid (EMD Chemical, Merck KGaA).

### Protein expression and purification

Kinase expression vectors were transformed into *E. coli* BL21 Star™ (DE3) cells (Life Technologies) in the presence or absence of pRSF1-PTP1B and/or the GroES–GroEL containing vector pGro7 (Takara Bio). Each of the three vector types contained a different antibiotic selection marker and origin of replication, enabling all three to be maintained within the same bacterial cell at any one time (pT7#3.3: Tet^r^, ColE1 origin, T7 promoter; pRSF-1b: Kan^r^, RSF1 origin, T7 promoter; pGro7: Cam^r^, pACYC origin, arabinose promoter).

Cells were cultured at 37°C, 220 rpm from a starting *D*_600_ (attenuance at 600 nM) of 0.075 in LB (Luria–Bertani) broth plus antibiotics. For pGro7-containing cultures, the addition of L-(+)-arabinose to a final concentration of 1 g/l was required to induce chaperone expression. At an average *D*_600_ of 0.35, the temperature was reduced to 18°C and expression of recombinant EphB proteins (and PTP1B, where present) was induced with 0.1 mM IPTG (isopropyl β-d-thiogalactopyranoside) at an average *D*_600_ of ~0.80 for 20 h. Cells were harvested by centrifugation at 12000 ***g***.

Cells were re-suspended in base buffer (40 mM Hepes, 500 mM NaCl and 1 mM TCEP [tris-(2-carboxyethyl)phosphine], pH 8.0) supplemented with 10 mM imidazole, 1 mg/ml hen egg white lysozyme (Sigma), 0.1 μl/ml Benzonase® Nuclease HC (Novagen) and EDTA-free Complete™ (protease inhibitor cocktail tablets; Roche). Cells were lysed using a 2.2 kW TS series cell lysis system (Constant Systems) at 25 kpsi. Insoluble material was removed by centrifugation at 35000 ***g*** for 60 min. Clarified supernatants were applied to 3 ml Ni-NTA (Ni^2+^-nitrilotriacetic acid) Superflow resin columns (Qiagen). The columns were washed with 10–50 CVs (column volumes) of base buffer supplemented with 25 mM imidazole. Bound proteins were eluted with base buffer supplemented with 0.5 M imidazole. Elution fractions were pooled and dialysed against dialysis buffer (40 mM Hepes, 0.5 M NaCl, 5 mM imidazole and 1 mM TCEP, pH 8.0) for 16 h at 4°C in the presence of His_6_-TEV protease (Life Technologies) to remove the His_6_-tag. The cleaved material was further purified by re-passing the dialysate over fresh Ni-NTA resin followed by a SEC (size-exclusion chromatography; Superdex S75; GE Healthcare) polishing step into a final containing 50 mM Mops, 50 mM NaCl and 1 mM DTT (dithiothreitol) pH 7.5. Peak fractions containing >95% pure EphB kinase as judged by SDS/PAGE were pooled, concentrated to 9.5 mg/ml and flash frozen in liquid nitrogen prior to storage at −80°C. All chromatographic manipulations were performed at +4°C.

### Determination of phosphorylation status

For detection of tyrosine phosphorylation of proteins from *E. coli* preparations, 0.5 μg affinity-purified kinase was analysed by Western blotting using 1:2000 anti-phosphotyrosine mouse monoclonal antibody (pY100; NEB Cell Signalling) with 1:1000 HRP (horseradish peroxidase)-conjugated rabbit-anti-mouse secondary antibody (Sigma) and detection using Supersignal West Femto ECL reagent (Thermo Scientific Pierce).

To obtain quantifiable phosphorylation data, EphB kinase samples at 1 mg/ml in crystallization buffer were loaded on to a Micromass LCT ES-TOF (liquid chromatography electrospray ionization time-of-flight) mass spectrometer, using a Waters 2790 HPLC as the inlet. 15 μg protein was injected for each measurement on to a Phenomenex Jupiter 5 m C5 300A column, 150×2.0 mm. Protein was eluted using a fast gradient [0–90% B over 45 min at 120 ml/min; eluent A was aqueous 0.1% TFA (trifluoroacetic acid), eluent B was 90% acetonitrile 0.1% TFA]. Electrospray mass spectrometer data were collected between 12 and 25 min post injection, and deconvoluted using MaxEnt1 software (Waters). Theoretical protein masses were calculated using the MassLynx™ software (Waters).

### Thermal stability analyses

Thermal unfolding measurements were conducted by CD using a Jasco J-810 Spectrapolarimeter with Peltier-controller. Proteins were rapidly defrosted and extensively dialysed against 50 mM sodium phosphate and 1 mM TCEP, pH 7.4. Protein concentrations were determined by attenuance at 280 nm using a Cary 300 Bio UV-Vis spectrophotometer and predicted molar absorption coefficient (ϵ). All CD measurements were conducted with 10 μM protein in a 1 mm path length non-demountable cuvette. Initial wavelength scans were performed at 20°C from 260 to 195 nm, with continuous scanning at 20 nm/min with a 1 nm bandwidth, 0.1 nm data pitch and a response of 2 s with standard sensitivity. Unfolding was monitored at 222 nm (α-helical response), with temperature scan from 20 to 80°C and a 1°C data pitch with a delay time of 60 s. The chosen response time was 4 s, with a 1 nm bandwidth and standard sensitivity. Three scans were performed for each protein. The primary data points (CD [mdeg] against temperature) were extracted and analysed within the Prism analysis package (version 5, GraphPad). The unfolding curves normalized and fitted to a six-parameter unfolding equation (Equation 1), adapted from [27] to obtain the *T*_m_ of unfolding and the Δ_U_*H*_app(Tm)_, the van't Hoff enthalpy.

(1) $$\begin{array}{ccc} & & Y_{\mathrm{obs}}\\ & & \frac{=\;\left\{\exp\left[-\Delta H_{m}\;\left(\;1-\frac{T}{T_{m}}\right)\;\;/\;\left(RT\right)\right]\;\left(a_{u}+b_{u}T\right)+\left(a_{n}+b_{n}T\right)\right\}}{\left\{1+\frac{\exp\left[-\Delta H_{m}\right]\left(1-\frac{T}{T_{m}}\right)}{RT}\right\}} \end{array}$$

where *T* is the temperature, *T*_m_ is the midpoint of the unfolding transition, Δ*H*_m_ is the change in enthalpy at the transition temperature (*T*_m_), *R* is the gas constant, *a*_n_ and *b*_n_ define the pre-transition and *a*_u_ and *b*_u_ define the post-transition regions of the curve. Two main parameters were extracted from this calculation: the *T*_m_ of unfolding and the Δ*H*_m_, which is Δ_U_*H*_(T)_ or Δ_U_*H*_app(Tm)_, the van't Hoff enthalpy.

### Solution-stability analyses

Chaotrope-induced protein unfolding was monitored by the change in intrinsic tryptophan fluorescence using Tecan XFLUOR4SAFIRE II plate-based scanning fluorimeter, with Greiner Black flat-bottom polystyrene 96- or 384-well plates. Two stock buffers were prepared from which a range of GdnHCl (guanidine hydrochloride) concentrations could be produced: fluorescence buffer A – 20 mM Hepes and 50 mM NaCl, pH 7.4; fluorescence buffer B – 20 mM Hepes, 50 mM NaCl and 6 M GdnHCl, pH 7.4. The final protein concentration used per well was 3 μM in a final volume of 15 μl (384-well) or 150 μl (96-well). Emission scans were conducted with an λ_ex_ of 295 nm and λ_em_ from 300 to 450 nm in 1 nm steps. Excitation and emission bandwidth was 5 nm, with a gain of 75 and integration time of 40 μs, lag time of 0 μs and temperature of 25°C±0.5°C. Peak tyrosine emission fluorescence for the folded proteins was judged to be 345 nm. Unfolding measurements were conducted with an λ_ex_ of 295 nm, λ_em_ of 345 nm and excitation and emission bandwidth was 2.5 nm, with a gain of 152 and integration time of 40 μs, lag time of 0 μs and temperature of 25°C±0.5°C. Unfolding curves were fitted to both two- and three-state unfolding models [28,29] using Prism (version 5; GraphPad) and Akaike information criteria to determine the probability of fit to the best unfolding model.

### Steady-state enzyme kinetics and small-molecule compound screening

Kinetic assays were based on *in vitro* phosphorylation of a generic tyrosine kinase substrate poly-(Glu:Tyr) (4:1, 20000–50000 Da; Sigma) in a stopped reaction format, using the ADP-Glo™ luminescent kinase assay kit (Promega). Reactions were performed in low-volume, flat-bottom white polystyrene 384-well plates in a total reaction volume of 4 μl; 2 μl/well of enzyme solution (50 nM EphB enzyme, 10 mM MgCl_2_, 100 mM Hepes, pH 7.5, 1 mM DTT and 0.02% v/v Brij-35) was mixed with 2 μl/well of ATP and substrate for a total of 140 min following the addition of enzyme, with time points at 0, 20, 40, 60, 80, 100, 120 and 140 min. The reactions were stopped by the addition of 4 μl of ADP-Glo™ Reagent 1 (Promega) with 40 min incubation, followed by 8 μl of kinase detection reagent for a further 60 min in the dark, before reading plates using a Pherastar plate reader (BMG Labtech) with a luminescence filter, at a read height of 14 mm and a 0.5 s integration time. All incubations were carried out at 21°C. The *K*_m_ for ATP was measured using a concentration range of 0–5 mM ATP at a fixed poly-(Glu:Tyr) concentration of 10 mg/ml. The *K*_m_ for poly-(Glu:Tyr) was measured using a concentration range of 0–10 mg/ml poly-(Glu:Tyr) at a fixed ATP concentration of 5 mM. Enzyme and substrate additions were made using a BioRAPTR (Beckman Coulter). Values for *K*_m_, *V*_max_ and *k*_cat_ were calculated using Prism analysis software (version 5; GraphPad) by Michaelis–Menten nonlinear regression analysis.

For the compound response testing, ATP and poly-(Glu:Tyr) were used at *K*_m_ for each kinase (Table 1) and the kinase reaction was incubated for 40 min. The compound concentration range was across 12 points. Compounds were dosed into assay-ready plates using 11 half-log intervals, followed by the 12th point, which was a whole log interval from the 11th (Echo 555; Labcyte). Each well was backfilled with the required volume up to 40 nl of 100% DMSO to ensure a final 1% DMSO concentration in the assay. Each plate contained at least 11 randomly distributed maximum and minimum controls. CMPD3 (compound 3, see Figure 4) was used to inhibit EphB constructs for the minimum control, with the exception of EphB1 and EphB3, where CMPD3 was replaced with an artificial minimum from full inhibition compounds from dose-response curves. For the maximum control, 40 nl pure DMSO was added to wells.

**Table 1:** Steady-state kinetic properties of the EphB enzymes Steady-state kinetic parameters of the four EphB enzymes measured in the luminescence peptide phosphorylation assay. Errors shown are calculated standard errors of three independent experiments (with the same protein batch for each enzyme).

	Steady-state kinetic data			
Enzyme	*K*_m_ ATP (*μ*M)	*K*_m_ poly(glutamic acid:tyrosine) (μg/ml)	*k*_cat_ (s^−1^)	*V*_max_ (nmol/min per mg)
EphB1	555±36	1137±125	0.80±0.02	1415±30
EphB2	751±80	1601±190	1.11±0.04	1802±67
EphB3	506±48	943±91	0.43±0.01	764±23
EphB4	497±39	1001±122	0.51±0.01	779±21
EphB3 C717G	659±67	1458±177	0.86±0.03	1533±46

### Determination of IC_50_

Using the maximum and minimum control wells as references for the 0% and 100% enzyme inhibition points, it was possible to calculate the effect of each compound on the kinase activity of each of the EphB constructs. For enzyme inhibition, nonlinear curve fit analysis within OriginLab™ software was used to fit dose-response curves, and was used to estimate the concentration of compound required to reduce the enzyme activity to 50%.

## RESULTS

### Recombinant kinase production

**Figure 1:**
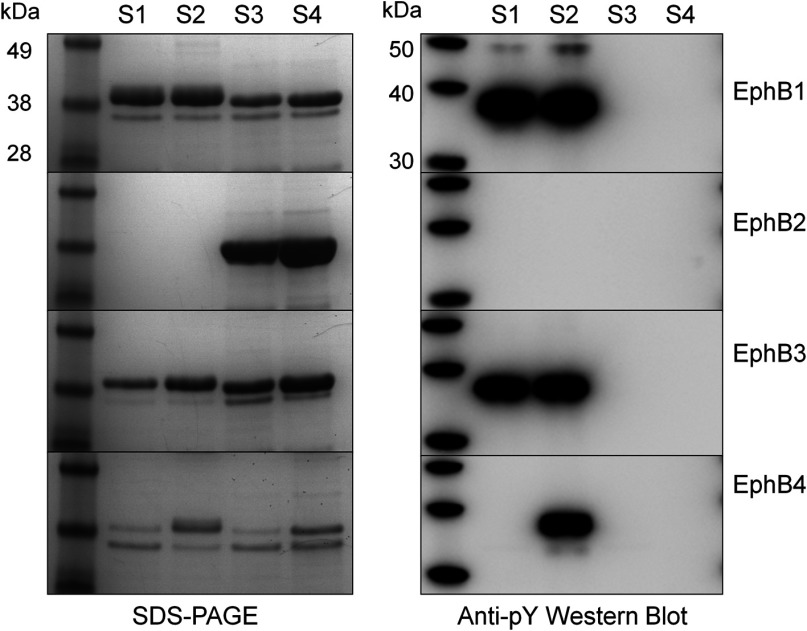
Recombinant expression analyses of EphB kinases in *E. coli* The left-hand panel shows SDS/PAGE gel analyses of hexahistidine affinity-purified samples of the four recombinant EphB kinases from the soluble fraction of *E. coli* cell lysates; 0.5 ml of culture equivalent loaded per lane. The right-hand panel shows an anti-phosphotyrosine Western-blot analysis of the same affinity purified samples; 0.5 μg of kinase loaded per lane. Expression strains: S1: host strain [BL21 Star (DE3)], S2: host strain plus pGro7, (GroEL/GroES), S3: host strain plus pRSF-PTP1B; S4: host strain plus pRSF-PTP1B and pGro7.

The EphB kinase domain construct boundaries chosen for this study were based on those used previously for EphB4 structure determination [30], and differ from those used for previous structural studies of EphB2 [31,32], as they do not contain the auto-inhibitory juxtamembrane region (Supplementary Figure S1 and Supplementary Table S1 available at http://www.bioscirep.org/bsr/033/bsr033e040add.htm). The recombinant *E. coli* expression of the four kinases was examined in the presence and absence of human PTP1B and/or the recombinant GroES–GroEL chaperone complex. The variation in soluble expression and phosphorylation state was marked between the four kinases as can be seen in Figure 1. EphB2 was found to be non-transformable, and therefore presumably toxic to the host *E. coli* strain used. This toxicity could be overcome through co-expression with PTP1B, resulting in high levels of soluble, purifiable material. EphB1 and EphB3 were both found to overexpress in the soluble fraction to >3 mg/l, despite being highly heterogeneously phosphorylated (Figure 1 and Supplementary Table S2 available at http://www.bioscirep.org/bsr/033/bsr033e040add.htm). For EphB4, the purifiable soluble expression level in the absence of GroES–GroEL was almost undetectable by SDS/PAGE (<0.1 mg/l), but was partially rescued by co-expression with GroEL/GroES, albeit at levels much lower than the other three kinases (Figure 1). EphB4, like EphB1 and 3, was also phosphorylated, with an average of two or three phosphorylations per molecule (Supplementary Table S2), all of which were removed through PTP1B co-expression. Using PTP1B (and GroES/GroEL, where required), each of the four kinase domains was expressed in a non-phosphorylated form, and was purified to >95% purity using a combination of IMAC (immobilized metal-ion-affinity chromatography) and SEC steps, allowing further characterization of the four proteins.

### Thermal stability

**Figure 2:**
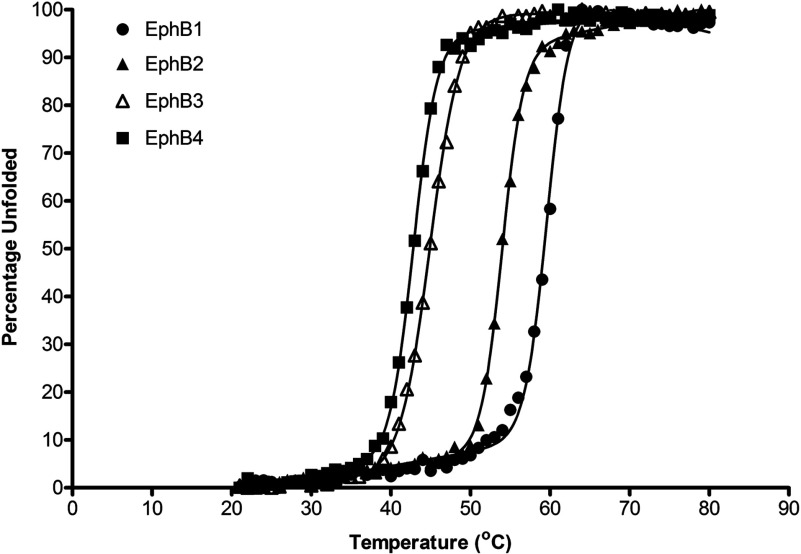
Thermal stability analyses using CD spectroscopy Representative unfolding transitions obtained for each of the four kinases from thermal denaturation experiments monitored using CD spectroscopy at 222 nm (α-helical response). Unfolding was conducted using 10 μM kinase at pH 7.4 under reducing conditions.

To investigate whether observed differences in soluble, recombinant expression levels in *E. coli* could be attributed to differences in stability between the isolated kinase domains, thermal unfolding events for each of the four kinase domains were studied using CD spectroscopy. Far UV wavelength scans were performed in phosphate buffer at physiological pH, and demonstrated very similar secondary structure profiles, as would be expected given the level of sequence and structural identity between the four domains (Supplementary Figure S2 available at http://www.bioscirep.org/bsr/033/bsr033e040add.htm). Strong α-helical signatures for all four proteins allowed temperature-dependent unfolding to be monitored at 222 nm. A two-state unfolding transition was observed for each protein over a 20–80°C range (Figure 2), enabling melting temperatures (*T*_m_, midpoint of unfolding) to be determined for all four kinases in their unphosphorylated forms (Table 2). A striking difference between the melting temperatures of the EphB kinase domains was observed, which coincidentally rank in order of their numbering, with EphB1 being the most stable, and a difference between apparent melting temperature for EphB1 and EphB4 of 16.9°C. The differences in thermal stability observed by CD unfolding were further confirmed using DSF (differential scanning fluorimetry) [33] (Supplementary Figure S3 available at http://www.bioscirep.org/bsr/033/bsr033e040add.htm).

**Table 2:** Thermodynamic parameters obtained from thermal and chaotrope unfolding CD thermal unfolding transition data were obtained and fitted as described. *n*>3, errors shown are calculated standard errors. For GdnHCl-induced unfolding monitored by intrinsic tryptophan fluorescence, Akaike information criteria probabilities were calculated by fitting the data to both two- and three-state unfolding equations [28,29].

	CD		GdnHCl Unfolding	
Enzyme	*T*_m_ (°C)	∆_U_H_(Tm)_ (kJ/mol)	Two-state probability (%)	Three-state probability (%)
EphB1	59.7±0.2	156.6±14.3	1.22	98.78
EphB2	53.9±0.2	154.2±16.5	<0.01	>99.99
EphB3	45.0±0.1	106.2±3.7	<0.01	>99.99
EphB4	42.8±0.2	129.3±10.7	<0.01	>99.99

### Chaotropic unfolding

**Figure 3:**
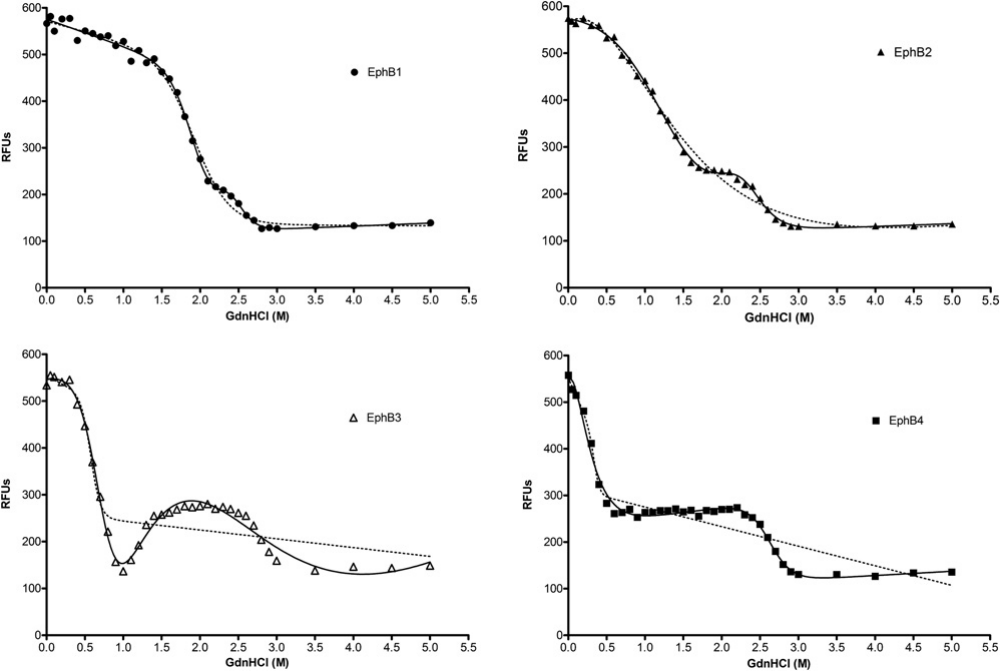
Chaotrope-induced denaturation of EphB kinase Unfolding EphB kinases in the presence of increasing GdnHCl was monitored by following the progressive quenching of internal tryptophan fluorescence (excitation 295 nm, emission 345 nm), as described. The *y*-axis shows relative fluorescence units measured at 345 nm averaged from five scans of three independent experiments. Curves were fitted to both two-state (broken line) and three-state (unbroken line) models [28,29], using Prism software (GraphPad Software, Inc.).

In an attempt to follow the unfolding events of each of the four kinases in greater detail, chaotrope-induced unfolding was performed. Internal tryptophan fluorescence of the four kinases was monitored at 345 nm in the presence of increasing GdnHCl concentration (Figure 3). Using this technique, a similar pattern of stability was observed to that of thermal unfolding. EphB1 appeared to tolerate a higher concentration of GdnHCl than the other enzymes before beginning to unfold. EphB2 appeared to start unfolding at a lower GdnHCl concentration, while EphB3 and EphB4 were both markedly less tolerant to chaotrope concentration. At a GdnHCl concentration of 3 M, a stable fluorescence minimum for all four kinase domains was reached, indicating complete unfolding. Each of the four proteins appears to fit more closely to a three-state unfolding model than a two-state model (Table 2), with evidence of a partially stable intermediate unfolding state at about 2 M GdnHCl, which appears more pronounced in EphB3 and EphB4, indicating a stable core common to each of the kinases. The most striking observation between the unfolding curves of the four proteins is the apparently ‘unfolded’ state observed at ~1 M GuHCl with EphB3: at this point, the tryptophan emission signal almost disappears and then reappears again at 1.5 M GuHCl.

### Kinetic profiling of the EphB kinase domains

An *in vitro* peptide phosphorylation assay was employed to investigate the intrinsic activity of each of the four isolated EphB catalytic domains. The unphosphorylated kinases were incubated with the generic tyrosine kinase substrate poly-(Glu:Tyr), and the level of substrate phosphorylation was monitored over time using an ADP-production luminescence assay. This assay was used to determine the comparative *K*_m_ of each kinase for ATP and substrate, as well as *k*_cat_ and *V*_max_ values (Table 1). These data showed that the affinities for ATP and substrate were similar for each of the enzymes, with the exception of EphB2, which was lower for both. EphB2 also exhibited the fastest turnover number, which was 30% greater than that of EphB1, and >2-fold faster than EphB3 or EphB4. Although there are differences observed in the *k*_cat_ and *K*_m_ values between the four enzymes, when comparing their specificity constants (*k*_cat_/*K*_m_), these are all found to be within two-fold of one another. We would therefore conclude that there is no significant difference between the substrate specificities of the isolated kinase domains as characterized within this assay system using the poly-(Glu:Tyr) substrate.

### Ligand-binding differences

**Figure 4:**
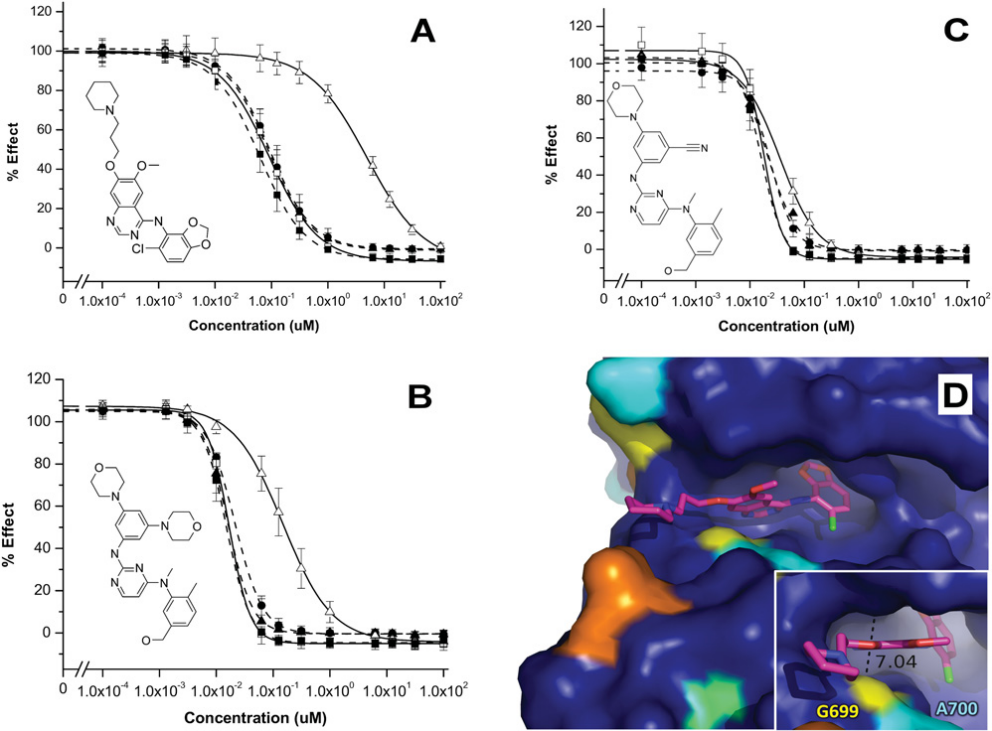
Dose-dependent inhibition of EphB enzymes using validated EphB4 kinase inhibitors An ADP-Glo™ assay was set up, as described. Inhibition by EphB4 (closed square ■; broken line), CMPD 1 (**A**) and 2(**B**) are shown to have similar activity for EphB1 (closed circle ●; broken line) as for EphB2 (closed triangle ▲; broken line) and EphB3CG (open square □; unbroken line), but is less potent against EphB3 (open triangle Δ; unbroken line) isoform. CMPD 3 (**C**), however, shows similar potencies for all isoforms. The *y*-axis represents luminescent response, where the minimum compound control signal was subtracted. For curve fitting, the mean max and min signals were used. Data shown are means±S.D. of three independent experiments. (**D**) The structure of the EphB4 active site (2VWU), with bound CMPD1 ligand (pink), rendered by amino acid conservation across the EphB family as detailed in Supplementary Figure S1. Insert shows location of Gly^699^ in EphB4 (equivalent to Cys^717^ in EphB3) relative to the position of CMPD1 at the mouth of the active site. The dashed line shows distance in Ångströms from Cα of Gly^699^ to the Cδ of Ile^621^ which forms the roof of the opening. PyMol was used to prepare the structure Figures (http://www.pymol.org).

The biochemical assay was used to screen a small panel of known tyrosine kinase inhibitors (Figure 4 and Supplementary Figure S4 available at http://www.bioscirep.org/bsr/033/bsr033e040add.htm). The panel included the known EphB4 inhibitors [CMPD1 (compound 1) from the anilinoquinazoline family [34]; CMPD2, a 2, 4-bisanilinopyrimidine [35]; and CMPD3, a cyano-substituted version of CMPD2], together with a selection of clinical tyrosine kinase inhibitors. The results show a range of potencies against the EphB kinases (Table 3). Interestingly, five of the seven compounds are markedly less potent against EphB3 than the other three kinases, with two exceptions: CMPD3 and Dasatinib [36]. This observation was confirmed by ITC (isothermal titration calorimetry), where the affinity of CMPD1 for each of the four kinases was measured (Supplementary Figure S5 available at http://www.bioscirep.org/bsr/033/bsr033e040add.htm). EphB3 has a much lower affinity for CMPD1 than the other three kinases (11.5 μM for EphB3 against sub-micromolar for the others). This difference is also demonstrated by a lower thermal stabilization effect of CMPD1 on EphB3 compared with the other three kinases in DSF compound-binding experiments (Supplementary Table S3 available at http://www.bioscirep.org/bsr/033/bsr033e040add.htm).

**Table 3:** Inhibition data for validated EphB4 and clinical tyrosine kinase inhibitors An ADP-Glo™ assay was performed and data normalized as described. The IC_50_ data are reported in μM. A single dose-response curve, for each compound, was plotted using normalized percentage effect for three independent experiments. The accuracy fit for each dose-response curve is shown by *R*^2^ (where 1=100% of points lie on fitted curve).

	EphB1		EphB2		EphB3		EphB3 C717G		EphB4	
Inhibitor	IC_50_	*R*^2^	IC_50_	*R*^2^	IC_50_	*R*^2^	IC_50_	*R*^2^	IC_50_	*R*^2^
CMPD1	0.091	0.994	0.088	0.978	4.958	0.98	0.09	0.983	0.062	0.99
CMPD2	0.021	0.998	0.016	0.992	0.149	0.991	0.017	0.999	0.015	0.994
CMPD3	0.023	0.992	0.021	0.988	0.036	0.997	0.018	0.991	0.017	0.997
Afatinib	5.443	0.971	2.488	0.954	>100	0.821	3.426	0.991	3.165	0.985
Dasatinib	0.01	0.999	0.007	0.993	0.007	0.993	0.008	0.995	0.006	0.981
Sorafenib	1.211	0.986	0.996	0.987	3.584	0.991	0.879	0.993	0.495	0.992
Sunitinib	5.025	0.992	2.903	0.954	37.27	0.98	2.808	0.988	3.47	0.993

As the binding mode of CMPD1 and CMPD2 in EphB4 has previously been determined by X-ray crystallography [34,35], we were able to use these structures together with a sequence alignment (Supplementary Figure S1), to look for differences between EphB3 and EphB4 in the binding region of these two compounds (Figure 4D). The most obvious difference was at Gly^699^ in EphB4 which is on the outer lip of the active site; this glycine is conserved in each of the EphB kinases except EphB3 where it is a cysteine (Supplementary Figure S1). As illustrated in Figure 4(D), the solubilizing group of CMPD1 extends out into the solvent channel past Gly^699.^ The presence of a cysteine in this position as found in EphB3 is likely to result in a steric clash with the solubilizing group, the position of which is constrained by the planar hinge binding group. This is likely to account for the lower potency of CMPD1 and related compounds with similar binding modes observed against EphB3 compared with other family members. Indeed, when this cysteine (Cys^717^) is mutated to a glycine, EphB3 demonstrates the same compound binding profile as the other three EphB kinases (Figure 4 and Table 3). It is worth noting at this point that the C717G mutant of EphB3 also alters the catalytic profile of the enzyme to make it more similar to EphB1 or EphB2 in terms of its *k*_cat_ and *V*_max_ (Table 1).

## DISCUSSION

### Disparities in recombinant EphB kinase expression profiles

Previous experimentation with the Eph-subfamilies has given rise to numerous interesting observations regarding their catalytic activation and auto-inhibitory mechanisms [31,32]. Related to these studies, observers have commented on the phosphorylation and toxicity issues regarding the recombinant expression of both Eph and the wider RTK family [32,37]. Wiesner et al. observed that bacterial expression of EphB2 (but not EphA4) resulted in toxicity, and thus implemented an inactivating mutation of the putative catalytic base to generate recombinant kinase in *E. coli* [32]. Predictably, these issues were not experienced when expressing the EphB2 kinase domain in an auto-inhibited catalytically repressed Tyr^604,610^ to phenylalanine double mutant form [31]. Our own in-house experiences with both the EphB2 and EphB4 subfamily members had led to similar observations (Green et al., AstraZeneca, unpublished work); while both kinases could be expressed in reasonable quantities in insect-cell expression systems, difficulties arose when attempting to produce material in *E. coli* for heteronuclear NMR studies. EphB2 could be bacterially expressed in a soluble form when present in a catalytically repressed or auto-inhibited form, but similar constructs of EphB4 only resulted in insoluble material, albeit at high levels.

The requirement for phosphatase co-expression to obtain homogeneous samples of protein kinases from recombinant expression systems has been previously described [37], and appears to be essential for EphB2 expression in *E. coli*. This is in stark contrast with EphB1, EphB3 and EphB4, which are also able to auto-phosphorylate, and are therefore active within the host cell, but whose activity does not appear to compromise *E. coli* growth. This disparity is likely to result from differences in substrate specificity between the EphB kinases, and potentially because EphB2 phosphorylation of one or more *E. coli* proteins is toxic to the cells. Supplementary Figure S1 shows the substrate-binding surface of the EphB4 kinase domain and the approximate binding orientation of the optimized Eph kinase synthetic peptide substrate EPHOPT, as defined by Davis et al. [38]. Although this surface is highly conserved between the EphB kinases, there are a few residues around this surface which are different in EphB2 and may afford some degree of selectivity, including Ala^700^, Ala^793^ and Ser^825^ which correspond to Ser^711^, Gln^799^ and Thr^831^ respectively in EphB2. These differences may allow the interaction of EphB2 with a different range of substrates and could account for the recombinant expression profile that we have observed. This agrees with the observation that EphB2 has a different substrate specificity to both EphB3 and EphB4 [38]. Additional experimentation attempt to determine whether this observation has relevance in a native human cellular context may increase our understanding of the specific roles of the different EphB kinases in terms of both normal physiology and disease.

For EphB4 the observation that we obtained transformants and cell growth in the absence of PTP1B makes toxicity an unlikely explanation for its low level of soluble expression. Also, as all four sequences had been codon-optimized for efficient transcription and translation, it would also seem unlikely that codon usage is the issue, although this cannot be ruled out. One potential explanation for this observation is lower intrinsic stability of this EphB4 construct compared with the other three proteins. The enhanced yields of all four kinases in the presence of GroES–GroEL indicates that the chaperonin complex is aiding the *in vivo* folding and/or solution stability of the Eph kinases and, in particular of EphB4; such effects are in line with previous claims about folding and solubilization effects of GroES–GroEL overexpression on other recombinant proteins [39].

### Intrinsic stability variation within the EphB kinase fold

One might expect two proteins that share a 14% difference in sequence identity (41 residues out of 294) to exhibit some degree of difference in stability profile, but the ~17°C difference observed between the melting temperatures of the isolated EphB4 and EphB1 kinase domains is dramatic. The disparity in *in vitro* stability is especially significant considering that these are two intracellular enzymes with very similar functions, and is highly likely to be the main contributing factor to the observed differences in their soluble expression yields from *E. coli*. It would be interesting to investigate whether these stability differences are a result of evolutionary pressure or of random substitutions that may or may not have an impact on the *in vivo* activity of these enzymes. Is this difference in stability functionally relevant, or is it just that the enzymes are stable enough for their role, and the differences in stability are just a result of evolved substrate or protein–protein interaction specificity? Taking into account the fact that these are isolated domains of much larger transmembrane receptor protein molecules, an interesting further study would be to examine whether the half-lives of the EphB receptors in native cells/tissues correlates with their intrinsic stability.

It is, at present, unclear whether the differences in stability and solubility have bearing on the activity profiles of the four isozymes. The two least stable enzymes, EphB3 and EphB4, do exhibit lower turnover numbers, which may relate in some way to their thermal stability or solubility. Although small variations were observed in their affinities for ATP and substrate, as well as their turnover numbers and efficiencies, we have not in this study examined in detail which residues contribute to the differences – an investigation that, although involved, might lead us to a better understanding of factors that contribute to kinase activity.

### EphB kinase compound profiling

The small selection of known EphB4 inhibitors and clinical tyrosine kinase inhibitors used in this study highlight the similarities in compound-binding profiles of EphB family members. The high level of sequence identity shared by the EphB family within the kinase domain means that, with the exception of EphB3, it might be very difficult to find isozyme-selective ATP-competitive inhibitors of the EphB family. To obtain selective EphB1, 2 or 4 kinase inhibitors, it may be necessary to exploit differences outside of the ATP-binding region, either by picking up long-range interactions or identifying alternative pockets that also modulate activity.

Conversely, the presence of Cys^717^ in EphB3 is unique among the Eph kinases and may afford the opportunity to design EphB3-specific kinase inhibitors. At present it is still unclear whether specific inhibitors of EphB3 catalytic activity might be of value in the clinic, they may, however, be useful in validating the role of EphB3 in the oncological diseases in which it has been shown to be up-regulated [40–43]. Although EphB3 kinase inhibitors have previously been described [44], it is unclear whether these inhibitors are selective enough to specifically target EphB3 against other EphB family members.

The reason for the lack of selectivity of Dasatinib is most likely a combination of its high potency and lack of sensitivity of the assay, which has a tight binding limit of approximately 25 nM. It is thought that the cyano substitution of CMPD3 may increase the potency against EphB3, owing to a specific interaction of the cyano moiety with Cys^717^, or a reduction in steric clash compared with the morphiline of CMPD2. In terms of its native biology, Cys^717^ may also have some influence on the substrate-binding specificity of EphB3, and is likely to have some *in vivo* significance in terms of regulation of activity.

### Conclusions

To conclude, the data we present highlight some dramatic and intriguing differences between members of this closely related family of protein kinases in terms of their physiochemical properties. It would appear from our findings that EphB3 is an outlier in terms of both its intrinsic folding mechanism and ligand-binding properties and that EphB2 may have a subtly different substrate-binding profile, which could have a biological significance. It is hoped that these observations will enable a greater biological understanding of this important class of receptors by facilitating production of recombinant protein tools, as well as potent and selective small molecules to aid mechanistic studies.
